# Bilateral simultaneous acute angle closure caused by sulphonamide derivatives: A case series

**DOI:** 10.4103/0301-4738.62657

**Published:** 2010

**Authors:** Sirisha Senthil, Chandrasekhar Garudadri, Harsha B L Rao, Rajat Maheshwari

**Affiliations:** L.V. Prasad Eye Institute, Kallam Anji Reddy Campus, L.V. Prasad Marg, Road No: 2, Banjara Hills, Hyderabad-500 034, India

**Keywords:** Bilateral acute angle closure, drug-induced, idiosyncratic response, sulphonamide

## Abstract

The sulphonamide group of drugs is implicated in bilateral acute angle closure (AAC) due to an idiosyncratic response. We report a series of three cases with bilateral AAC caused by different sulphonamide derivatives, their presentation and management.

Bilateral simultaneous secondary acute angle closure (AAC) is rare and has been reported secondary to drugs,[1] general anesthesia,[2] snake bite,[3] microspherophakia[4] and Vogt-Koyanagi-Harada syndrome.[5] Amongst drugs, sulphonamide and its derivatives have been documented to cause transient myopia, ciliary body edema, uveal effusions and anterior rotation of the lens-iris diaphragm causing secondary angle closure.[6] We report a series of three patients who were on various sulphonamide derivatives and presented with bilateral AAC.

## Case Reports

### Case 1

**Figure 1:**
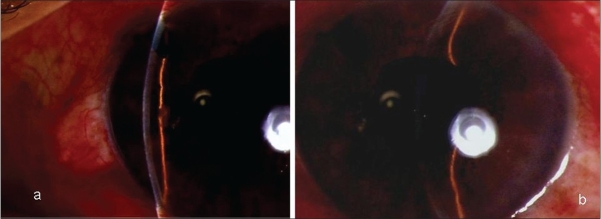
(a) (Right Eye): Slit-lamp photograph showing shallow anterior chamber with posterior chamber intraocular lens and patent peripheral iridotomy (b) (Left Eye): Slit-lamp photograph showing shallow anterior chamber with posterior chamber intraocular lens

**Figure 2:**
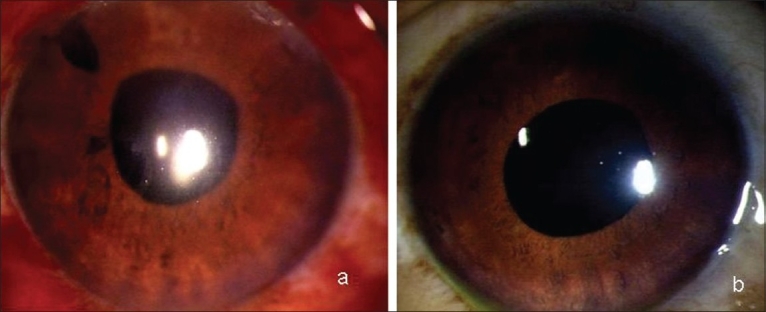
Slit-lamp photograph (a) (Right Eye) and (b) (Left Eye) showing deep anterior chambers after resolution of the condition

A 60-year-old male was referred to us with a history of having undergone cataract surgery in left eye (L/E) two days earlier and in right eye (R/E) one month ago. He complained of severe pain and decreased vision in both eyes for two days. He had undergone phacoemulsification under topical anesthesia in both the eyes one month apart and the postoperative period after the first eye surgery was uneventful. He presented to the treating physician with bilateral flat anterior chambers and high intraocular pressure (IOP) four hours after his cataract surgery and was diagnosed and treated as malignant glaucoma with intravenous (IV) mannitol 20% (1-2 g/kg body weight), tab acetazolamide 250 mg thrice daily and timolol maleate 0.5% eye drops twice daily in both eyes (B/E), peripheral iridotomy in R/E and YAG hyloidotomy in R/E and posterior capsulotomy in L/E. On examination here, visual acuity was finger counting at half meter in R/E and one meter in L/E. There was bilateral lid edema, conjunctival chemosis, corneal edema, shallow anterior chambers (ACs) [Fig. 1a and b], mid-dilated pupils, patent peripheral iridotomy in R/E, in-the-bag posterior chamber pseudophakos with opening in the posterior capsule. IOP was 72 mmHg in R/E and 68 mmHg in L/E. Fundus was unclear. B-scan Ultrasonography revealed annular cilio-choroidal effusion in B/E. A possible drug reaction was suspected and the systemic drug list was reviewed. He had been a diabetic for 15 years, medically controlled. He was given tab. acetazolamide 250 mg prophylactically after both his cataract surgeries. Acetazolamide, a sulphonamide derivative was suspected as the possible cause and was discontinued. Topical antiglaucoma medications along with atropine, topical steroids and antibiotics were continued. Over the next two weeks his condition improved. Five weeks following the initial surgery his vision was 20/20, N6 with correction and IOP 21 mm Hg in R/E and 20 mm Hg in L/E without medications. ACs were quiet with clear corneas in both eyes [Fig. 2a and b] and gonioscopy showed open angles in both eyes. Fundus examination revealed healthy optic discs with 0.5:1 cup and resolved choroidals in both eyes.

### Case 2

**Figure 3:**
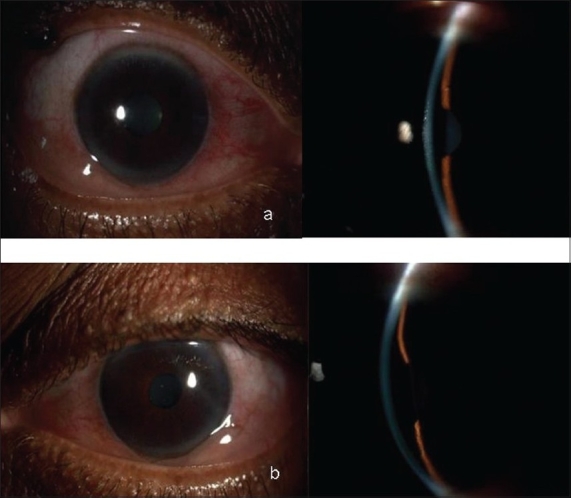
Slit-lamp photographs (a) (Right Eye) and (b) (Left Eye) showing congested eyes with shallow anterior chambers

**Figure 4:**
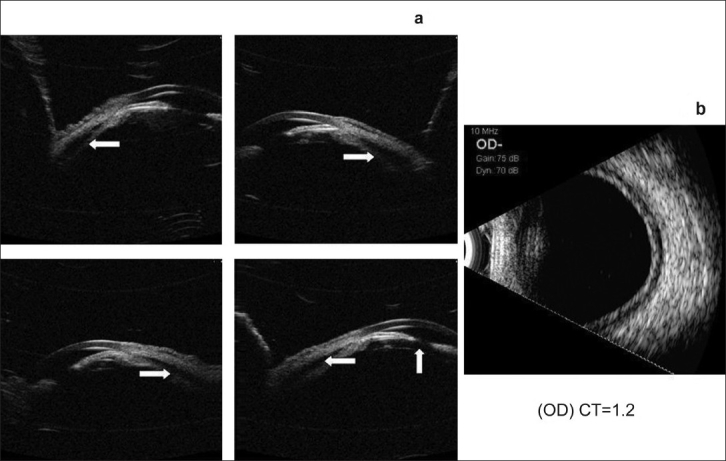
Ultrasound biomicroscopy (a) Right Eye showing 360° ciliochoroidal detachments with angle closure and no pupillary block (b) ultrasound B-scan showing shallow choroidal detachment

**Figure 5:**
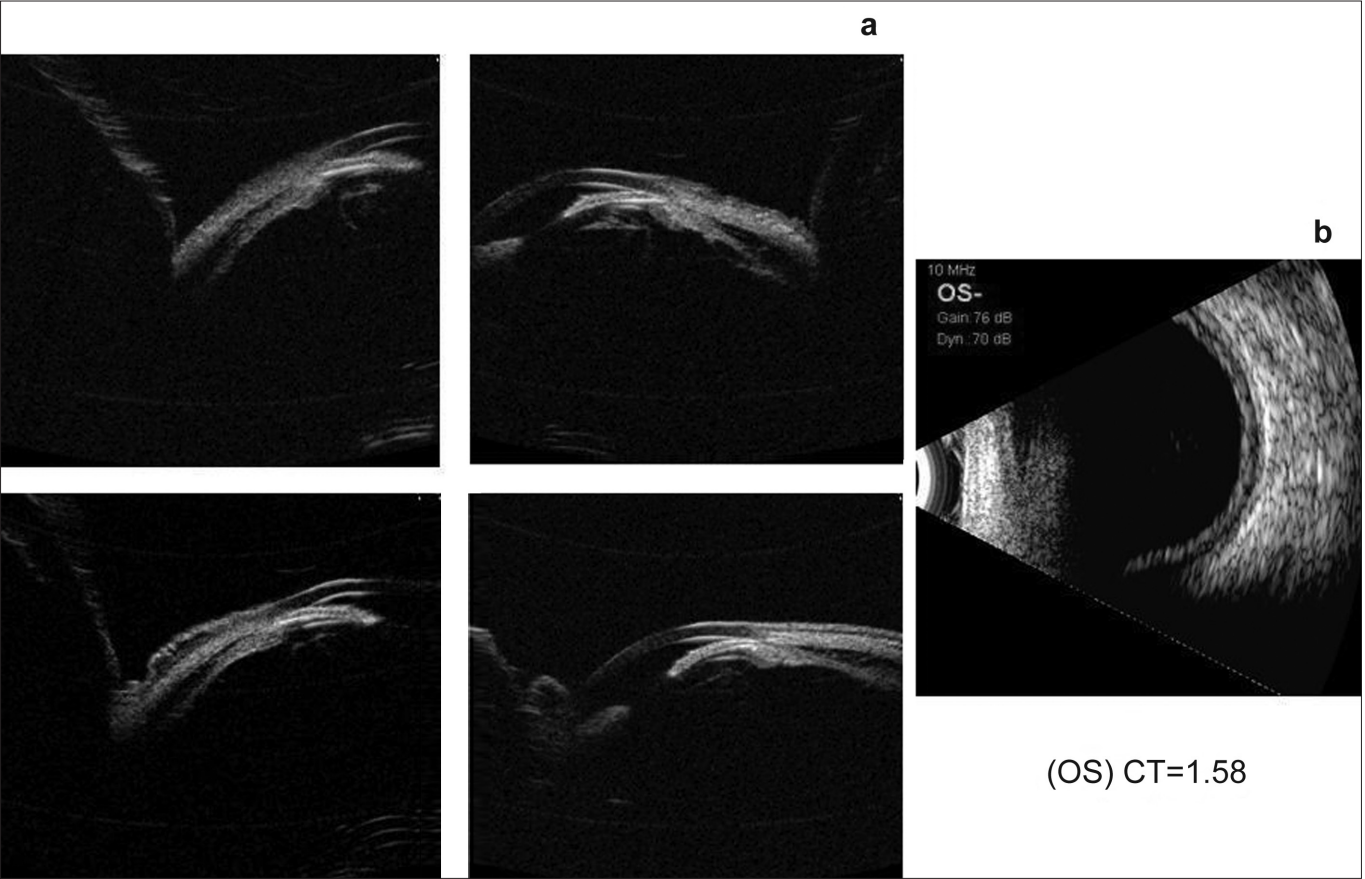
Ultrasound biomicroscopy (a) Left Eye showing 360° ciliochoroidal detachments with angle closure (b) ultrasound B-scan showing shallow choroidal detachment

**Figure 6:**
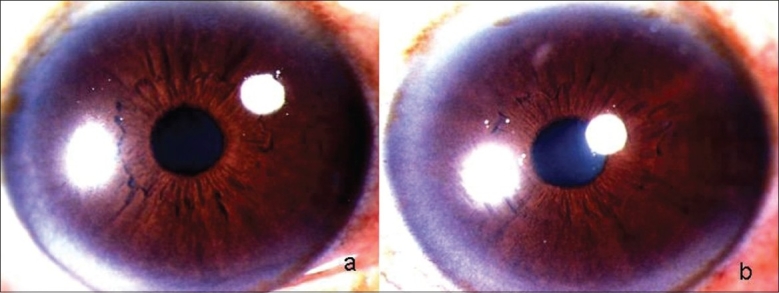
Slit-lamp photographs showing normal anterior segments in Right Eye (a) and Left Eye (b) after complete resolution of the condition

**Figure 7:**
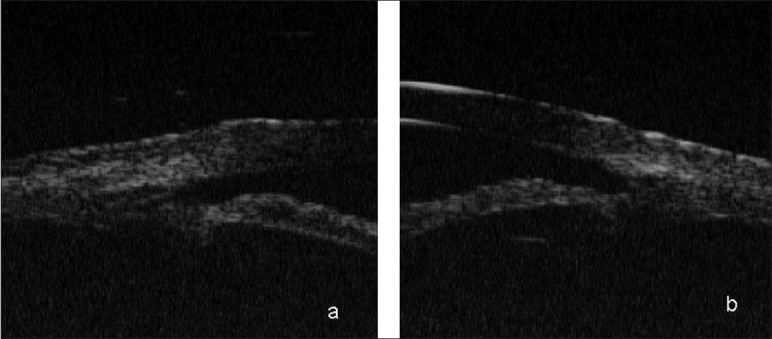
Ultrasound biomicroscopy showing wide open angles after the resolution of the condition

A 53-year-old woman presented with sudden painful decrease in vision in B/E and severe headache for 12 h. She was a known diabetic and hypertensive on insulin, amlodepine 5 mg for the past two years and indapamide 2.5 mg for one month. Her random blood sugar was 180 mg/dl. On examination, her vision was 20/60p, N36 R/E and 20/60p, N24 in L/E with a myopic shift of one diopter (D) in B/E. In B/E there was lid edema, conjunctival chemosis, shallow ACs [Fig. 3a and 3b], closed angles on gonioscopy, round and sluggishly reacting pupils and clear lens. The IOP was 57 and 52 mm Hg in R/E and L/E respectively. Fundus examination revealed 0.3 cupping in B/E. An ultrasound biomicroscopy (UBM) examination revealed bilateral 360° ciliochoroidal effusion with forward rotation of ciliary body and narrowing of ciliary sulcus [Figs. 4 and 5]. With these clinical features a drug-induced angle closure was suspected. Indapamide, a sulphonamide-derived thiazide diuretic was suspected to be the probable cause. After discussion with her physician, Indapamide was discontinued. She was started on IV mannitol and topical antiglaucoma medications. By Day five her visual acuity with her presenting glasses was 20/20, N6 in both eyes, IOP was 13, 14 mm Hg in R/E and L/E respectively with normal anterior segment [Fig. 6a and b] and complete resolution of ciliochoroidal effusion with wide open angles on UBM [Fig. 7a and b]. She was advised to discontinue all ocular medications.

### Case 3

A 28-year-old woman presented with sudden decrease in vision and pain in B/E for one day. She had been recently diagnosed to have migraine and was started on tab. topiramate 50 mg for four days. On examination her vision was 20/200 both eyes, improving to 20/30 with -5.0 D sphere correction, both eyes showed congested conjunctivae, clear corneas, shallow ACs, clear lens and round reacting pupils. IOP was 34 mm Hg in B/E. Gonioscopy showed closed angles and an undilated fundus examination showed 0.4 cup with healthy rim in B/E. With sudden decrease in vision, myopic shift in refraction and bilateral AAC, a diagnosis of topiramate-induced secondary angle closure was made. She was advised to discontinue topiramate and was treated with IV mannitol and timolol maleate 0.5% drops. Three days later her unaided vision was 20/20 in B/E with IOP 14 mm Hg and wide open angles on gonioscopy. Timolol was discontinued.

## Discussion

Drug-induced uveal effusions have been reported with sulphonamides.[6] All three patients were on commonly used sulfonamide derivatives prior to presentation. Exact mechanism of choroidal effusion with these drugs remains unclear. Effusion in only a few patients taking these drugs suggests a possible idiosyncratic reaction.[6] Acute secondary angle closure and acute myopia are caused by the associated ciliochoroidal detachment with forward shift of the iris lens diaphragm.[1]

The fact that the condition resolved after stopping the suspected medication indicates a causal relationship. The best method to prove causality is to re-challenge. However, re-challenge may not be ethical and might sometimes fail to reproduce the event.

It is rather paradoxical that acetazolamide, an antiglaucoma medication can cause secondary angle closure and rise in IOP.[7] Since acetazolamide was not suspected as the cause in our first case, it was misdiagnosed as malignant glaucoma and the drug was continued. Idiosyncratic drug reaction, which is the suspected mechanism in this condition, is not dose-dependent. The most prevalent hypothesis is the hapten hypothesis, which says that the reactive drug metabolites bind to the proteins and these altered proteins are perceived as foreign and induce an immune response. Developing the reaction with a single dose is uncommon. In our patient probably the first dose was a sensitizing dose and the second dose precipitated the condition. In Case 1, until patient was on oral acetazolamide (which was continued even after AAC) despite antiglaucoma medications, the IOP remained high. It was only after discontinuation of acetazolamide the condition improved which indirectly proved to be causal.

To our knowledge there is no report of bilateral drug-induced angle closure caused by indapamide, an antihypertensive which is a sulphonamide derivative. However, there is a report of acute transient myopia caused by indapamide.[8] Topiramate, a commonly used antimigraine and antiepileptic drug has been reported to cause acute myopia and angle closure.[9,10] In all our cases, stopping the offending agent and controlling the IOP medically resulted in rapid resolution of the bilateral angle closure without any intervention.

This case series highlights the fact that drug-induced angle closure should be suspected in any patient with features of bilateral AAC, myopic shift of refractive error and annular ciliochoroidal detachment. UBM is helpful in the diagnosis of this condition. Apart from IOP-lowering measures, a review of systemic medications and discontinuation of the most probable causative agent should be done. A high index of suspicion will help manage drug-induced bilateral acute angle closures appropriately with quick and complete visual recovery.
